# Two mitochondrial genomes of Taiwanese rhinoceros beetles, *Oryctes rhinoceros* and *Eophileurus chinensis* (Coleoptera: Scarabaeidae)

**DOI:** 10.1080/23802359.2021.1948364

**Published:** 2021-07-06

**Authors:** Chung-Te Cheng, Ming-Luen Jeng, Jing-Fu Tsai, Chun-Lin Li, Li-Wei Wu

**Affiliations:** a Department of Life Science, Tunghai University, Taichung, Taiwan, R.O.C.;; b Department of Biology, National Museum of Natural Science, Taichung, Taiwan, R.O.C.;; c The Experimental Forest, National Taiwan University, Taipei, Taiwan, R.O.C.

**Keywords:** Mitochondrial meta-genomics, dung beetles, mitochondrion, coprophagous, phytophagous

## Abstract

Two mitochondrial genomes of the dynastine beetles, *Oryctes rhinoceros* (Linnaeus, 1758) and *Eophileurus chinensis* (Faldermann, 1835), were assembled *via* high-throughput sequencing (HTS). Each of the mitogenomes has 37 genes, showing standard gene order and annotation as the other insects, except for the transfer genes, presenting tQ-tI-tM order. To examine their phylogenetic positions, 118 public mitogenomes of Scarabaeidae were used to infer a ML tree. Overall, our scarabaeid phylogeny reveals clear relationships with high nodal supports, and the two rhinoceros beetles are both grouped with the subfamily Dynastinae. The feeding habit of the two clades seems to represent coprophagous and phytophagous types. However, polyphyletic relationships were observed in the subfamily Melolothinae and in the tribes of Onthophagini and Oniticellini. Further systematic revision is needed.

The subfamily Dynastinae MacLeay, 1819 (Coleoptera: Scarabaeidae) is a fascinating beetle group, comprising eight tribes and over 1500 species (Bouchard et al. 2011; Beutel and Leschen 2016). The exaggerated ornaments of the Dynastinae males (especially Dynastini) are well discussed for sexual selection (Ito et al. 2013), and many enthusiasts are also obsessed with their morphological diversity. However, the high-level relationships of Dynastinae remain largely unknown, and even a few studies have pointed out polyphyletic relationships in the tribe-level (Hunt et al. 2007; Gunter et al. 2016; Paucar-Cabrera and Moore 2018; Šípek et al. 2016; Song and Zhang 2018; Eberle et al. 2019; Filipović et al. 2021). More robust phylogenetic relationships of Dynastinae are needed, however, only one complete and one partial mitogenomes of the Dynastinae were published in GenBank (accessed on 15 March 2021). To increase mitogenomic references, two dynastine beetles from Taiwan were sampled in this work.

The specimen of *Oryctes rhinoceros* (Linnaeus 1758) was collected from Jiji Township, Nantou County (coordinate: N:23.8282, E:120.8013; DNA code: 20LW12002). The species, *Eophileurus chinensis* (Faldermann 1835) was obtained from Jinfeng Township, Taitung County (coordinate: N:22.6362, E:120.9718; DNA code: 20LW12003). The genomic DNAs were extracted from head tissues using Gentra Puregene Tissue Kit (Gentra Systems, Minneapolis, MN) and then restored with 15 μL of sterile H_2_O. The concentrations were both over 25 ng/μL, measured by using Qubit dsDNA HS Assay kit (Thermo Fisher Scientific, Waltham, MA), and then all the extracts were sheared into 200–600 bp to construct NGS library using NuGEN Ovation Ultralow library System (NuGEN Technologies, San Carlos, CA) for high-throughput sequencing (HTS) *via* Illumina Miseq platform.

There are 2,021,079 (*O. rhinoceros*) and 2,899,748 (*E. chinensis*) reads after removing out low-quality regions (below Q20). Each HTS dataset was *de novo* assembled with 97% similarity using CLC Genomics Workbench and megahit 1.2 (Li et al. 2015), then mapping with a 827-taxa mitogenomic dataset (Supplementary file 1) to filter out mitogenome-like sequences (set to 70% similarity). The sequences were corrected and edited *via* combining these two assembled results using Sequencher version 4.10 (GeneCode, Boston, MA). Finally, two mitogenomic sequences were obtained: *O. rhinoceros* has 15,339 bp in length (average coverage = 303–312) and *E. chinensis* has a complete mitogenome with 16,624 bp in length (average coverage = 56–86). Their gene regions and annotation were analyzed *via* MITOS2 website (Bernt et al. 2013), and the gene positions were checked against the references (accession numbers: MT457818, KU739467, and NC_023246). The newly obtained mitogenomes both have complete 13 protein-coding genes (PCGs), 2 ribosomal RNA genes, and 22 transfer RNA genes, but the control region of *O. rhinoceros* is partial. Their gene order and direction have standard order in insects, except for the order of three tRNAs, presenting ‘tQ-tI-tM’ order instead of ‘tI-tQ-tM’ (Boore 1999).

Combining with two newly sequenced mitogenomes, a total of 118 scarabaeid mitogenomic sequences were obtained from GenBank. Each mitochondrial gene was aligned using MUSCLE (Edgar 2004), implied in MEGA-X (Kumar et al. 2018). PCGs were aligned with codon positions, whereas tRNAs and rRNAs were aligned directly with default setting. All the sequences were concatenated, and the aligned dataset is 15,796 bp in length. For inferring phylogeny, the *Hybosorus* sp. (Hybosoridae) was set as outgroup and the best partition scheme (eight partitions: *atp6*, *nad3*; *atp8*, *nad2*, *nad6*; *cob*, *cox1*, *cox2*, *cox3*; *nad1*; *nad4*, *nad4l*, *nad5*; *rrnL*; *rrnS*; tRNAs) was selected using PartitionFinder version 2.1.1 (Lanfear et al. 2012). The ML phylogeny was reconstructed using IQ-TREE (Nguyen et al. 2015), and the nodal supports were evaluated by 1000 replicates of bootstrapping.

Overall, our phylogenetic relationships (Figure 1; Supplementary file 2) show concordant with previous studies (Gunter et al. 2016; Tarasov and Dimitrov 2016; Song and Zhang 2018), but our results provide more strong supports on branch nodes. The subfamily Dynastinae is monophyletic, and two major clades, associated with feeding habit, are presented: phytophagous group (including Rutelinae, Dynastinae, Cetoniinae, and Melolothinae) and coprophagous group (including Aphodiinae and Scarabaeinae, known as ‘dung beetles’) (Mckenna et al. 2015; Gunter et al. 2016; Eberle et al. 2019). However, some polyphyletic relationships are observed: the tribe Oniticellini was nested within Onthophagini (Figure 1), while the subfamily Melolothinae is polyphyletic, similar to previous studies (Mckenna et al. 2015; Gunter et al. 2016; Eberle et al. 2019).

**Figure 1.**
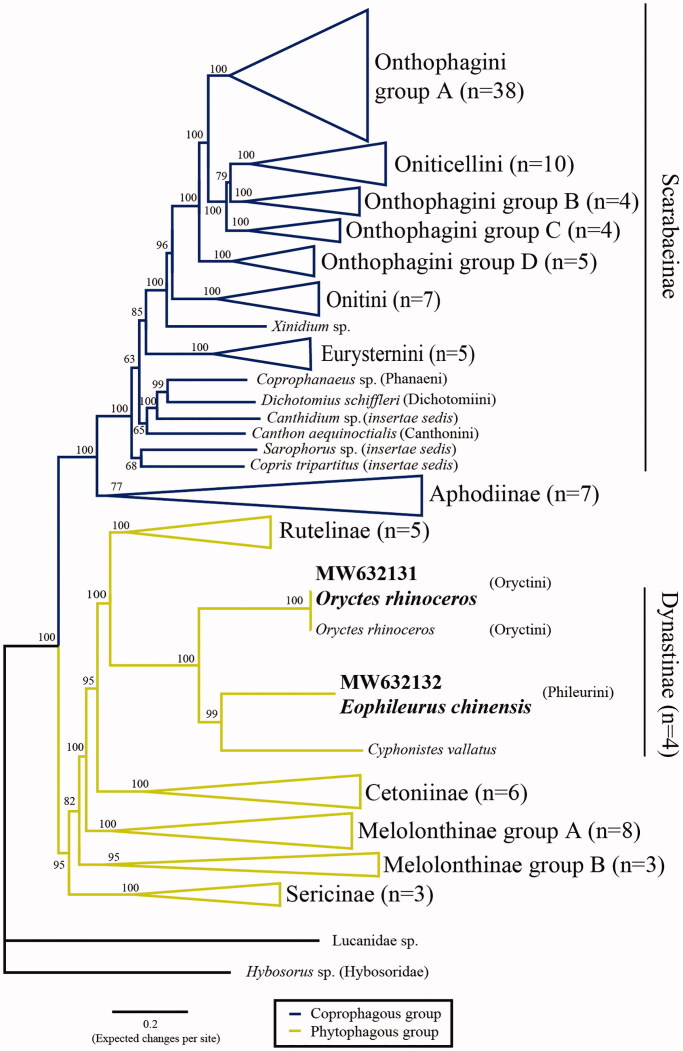
The ML topology of the Scarabaeidae using IQ-TREE. Hybosorus sp. and Lucanidae sp. were set as outgroups. Two newly sequenced genomes were deposited to GenBank and labelled in bold characters. The tribal concepts of dung beetle were based on Tarasov and Dimitrov (2016).

## Data Availability

The data that support the findings of this study are available in National Center for Biotechnology Information (NCBI) at https://www.ncbi.nlm.nih.gov/nucleotide/, reference numbers: MW632131, and MW632132. The raw sequence data was deposited in SRA database, accession number PRJNA735922. Supplementary files are available in figshare at https://figshare.com/articles/dataset/Supplementary_file_rar/14195876. Specimens of *O. rhinoceros* and *E. chinensis* were both deposited at National Museum of Natural Science, Taichung (contact person: Jing-Fu Tsai, email: jftsai.nmns@gmail.com) under the vouchers of NMMS ENT 8348-1 and NMNS ENT 8348-2, respectively.
